# Risks and consequences of TB and its prevention in cost-utility analyses among immigrants: a systematic review

**DOI:** 10.5588/ijtldopen.25.0355

**Published:** 2025-10-10

**Authors:** S.D. Robayo, C.M. Tansey, K. Romanowski, J.R. Campbell

**Affiliations:** ^1^ Department of Experimental Medicine, McGill University, Montreal, Canada;; ^2^ Respiratory Epidemiology and Clinical Research Unit, Centre for Outcomes Research and Evaluation, Research Institute of the McGill University Health Centre, Montreal, Canada;; ^3^ Department of Global and Public Health, McGill University, Montreal, Canada;; ^4^ Tuberculosis Services, BC Centre for Disease Control, Vancouver, Canada;; ^5^ McGill International TB Centre, Montreal, Canada;; ^6^ Department of Medicine, McGill University, Montreal, Canada.

**Keywords:** tuberculosis, TB infection, infectious disease, economic evaluation, public health, global health

## Abstract

**BACKGROUND:**

In low-TB incidence countries, foreign-born populations bear a disproportionate share of the TB burden. Cost-utility analyses of TB preventive treatment (TPT) programs among immigrants, however, have yielded divergent conclusions. We conducted a systematic review to evaluate how studies have considered the risks and consequences of TB disease and TPT.

**METHODS:**

We searched PubMed and Embase for studies published from 1^st^ January 2004 to 25^th^ March 2025. We included modeling studies measuring health utility with quality-adjusted life years (QALYs) and evaluated TPT among immigrants to low TB incidence countries. Using a narrative synthesis, we examined how studies considered risks and consequences of TB disease and TPT and their impacts on health utility.

**RESULTS:**

Of the 5,142 records screened, 14 studies met the inclusion criteria. Major adverse events (AEs) were the most frequently considered consequence of TPT with estimated risk ranging from 0% to 6% and mean associated annual disutility from major AEs was 0.017 QALYs, which varied substantially (coefficient of variation [CV)]: 1.2). All studies considered health disutility due to TB disease, with annual disutility ranging from 0.04 to 0.2 (mean: 0.11, CV: 0.4).

**CONCLUSIONS:**

There is wide variation in how risks and consequences of TPT and TB disease are considered in studies evaluating TB infection treatment programs.

TB remains a major global health challenge and the leading cause of death from a single infectious agent, claiming an estimated 1.25 million lives in 2023.^1^ While the burden is concentrated in high-incidence countries, TB continues to pose a challenge in many low-incidence countries (defined as <30 notifications per 100,000 population per year).^2,3^ In these countries, the TB burden is disproportionately borne by immigrant populations, with most diagnoses stemming from progression of TB infection (TBI) acquired prior to migration.^4^ Given this epidemiological context, TB preventive treatment (TPT) has become an essential strategy to reduce TB disease, making these programs a focus of growing interest.^5^ However, only a few countries, including Norway and the USA, have implemented broad programs to detect and treat TBI among new immigrants.^6–9^ The limited global uptake of such programs may reflect conflicting conclusions about their cost-effectiveness. For example, economic evaluations conducted in Canada, Australia, and Sweden^10–12^ have found these programs to be poorly cost-effective, while similar analyses in the USA suggest they could indeed be cost-effective.^13,14^ These divergent conclusions across models considering similar interventions in similar populations raise important questions about the factors driving these discrepancies.

Understanding the reasons behind these conflicting findings requires a closer look at how the economic models are constructed and the parameters included. Some model parameters, such as TBI prevalence, risk of progression to TB disease, and costs, are population- and setting-specific. Others, such as TPT acceptance and completion, reflect programmatic differences and are reasonable to vary across models. However, regimen-specific parameters, including the risks of adverse events and hospitalization rates, and health utility estimates, should be consistent across studies. Moreover, studies should be consistent in the types of risks considered, as selective inclusion or omission may bias results. In this study, we sought to investigate how cost-utility analyses of TBI testing and treatment programs in immigrant populations in low-incidence countries have considered the risks and consequences of TPT and TB disease to support more standardized parameterization moving forward.

## METHODS

This review followed the PRISMA reporting guidelines^15^ and was registered prospectively (CRD420251015358). We systematically searched PubMed and Embase from 1^st^ January 2004 to 25^th^ March 2025, using a broad search strategy without language restrictions **(**see Supplementary Data Table S1**).** Keywords included ‘tuberculosis’, ‘cost-utility analyses’ and ‘quality adjusted life year’. This timeframe was selected to align with contemporary modelling approaches, TPT regimens, and increasing policy interest in expanding TBI testing and treatment programs for immigrants to low-incidence countries. We also reviewed reference lists of all identified studies and relevant systematic reviews identified in our search to identify other potentially eligible studies. Eligible studies met the following criteria: (1) were economic modeling studies; (2) measured health utility using quality adjusted life years (QALYs); (3) evaluated the use of TPT with or without TBI diagnostic testing (e.g., tuberculin skin testing [TST], interferon-gamma release assay [IGRA] or TB antigen skin test); and (4) modeled a population of immigrants to a low-incidence country, defined as having fewer than 30 notifications per 100,000 population per year. We excluded studies that reported on broad TBI testing and treatment programs of which immigrants were a subgroup if they did not provide stratified information on the immigrant population. Studies restricted to specific high-risk subgroups, such as TB contacts or individuals screened only after risk-factor diagnosis post-migration were also excluded. Titles and abstracts were uploaded to Covidence *(Veritas Health Innovation, Australia)*, a web-based platform for managing systematic reviews. Two reviewers (SDR and CT) independently screened all records. Any study selected by either reviewer were advanced for full-text review, which was conducted independently by the same reviewers, with disagreement resolved by a third reviewer (JRC).

### Data extraction

A standardized extraction form was developed *a priori* to ensure consistency. A key component of the form was to the capture of potential consequences related to TPT and TB disease that could influence health utility. For TPT, we considered: the impacts of TBI or act of taking treatment (e.g., psychological impact of diagnosis, pill burden), adverse events of treatment, hospitalization, and death. For TB disease, we considered: the impact of disease itself (e.g., symptoms), adverse events of treatment, hospitalization, death, and post- TB sequelae. The form was developed by SDR and JRC and piloted on five studies before finalization. Two reviewers (SDR and CT) extracted data from the full-text articles independently. A third reviewer (JRC) verified the consistency of the data extraction. We extracted data on study and population characteristics, diagnostic tests and TPT regimens. For pre-specified health states (e.g., healthy, TB, TBI, TPT, post- TB, adverse events, hospitalization, death), we collected information on associated health utilities, including their source and duration of impact. We also extracted the probabilities of specific events (e.g., death, adverse events, hospitalization) and the definitions used in each study (Supplementary Data Table S2). Three authors were contacted to provide additional information and only one responded.

### Outcomes

The primary outcomes were: (1) the probability of events associated with TB disease and TPT, including major and minor adverse events, hospitalization, and mortality; and (2) health disutility associated with TB disease and its treatment, post- TB health states, TBI and its treatment, hospitalization, and major and minor adverse events. As we anticipated health disutility would vary across studies due to differences in the duration of their impact, we standardized all health disutility to be on an annual scale. We used study specific definitions of major and minor adverse events related to TB treatment and TPT, however when unclear, we assumed major adverse events were those resulting in the discontinuation of treatment.

### Quality assessment

Two reviewers (SDR and JRC) independently assessed the quality of the included studies, using three domains from the Checklist for Health Economic Quality Evaluations (CHEQUE 2023) framework,^16^ selected for their relevance with the objectives of our review. We focused on the following attributes: (1) whether all relevant events and processes in TBI and TB disease were considered; (2) whether parameters were informed by the best available, high-quality evidence (such as systematic reviews or local data) to inform the parameters; and (3) whether health utility parameters were evidence-based and reflective of the population being modeled. Each attribute was graded according to the checklist scoring criteria and given an overall percentage grade.

### Analysis

Given the nature of the data and modeling approaches, we conducted a narrative synthesis. We synthesized how each study modeled the target population, their characteristics, and the interventions evaluated. We examined which risks and consequences were considered and how they were defined. Where relevant, we reported ranges, averages, and coefficients of variation for probabilities and health utility or disutility. We focused our synthesis on the two most common consequences considered: major TPT-related adverse events and TB disease. Finally, we mapped the original sources used in each study to parameterize health disutility to understand differences in model parameterization.

### Ethical statement

This study did not require ethics approval as it relied on previously published data.

## RESULTS

**Figure 1.**
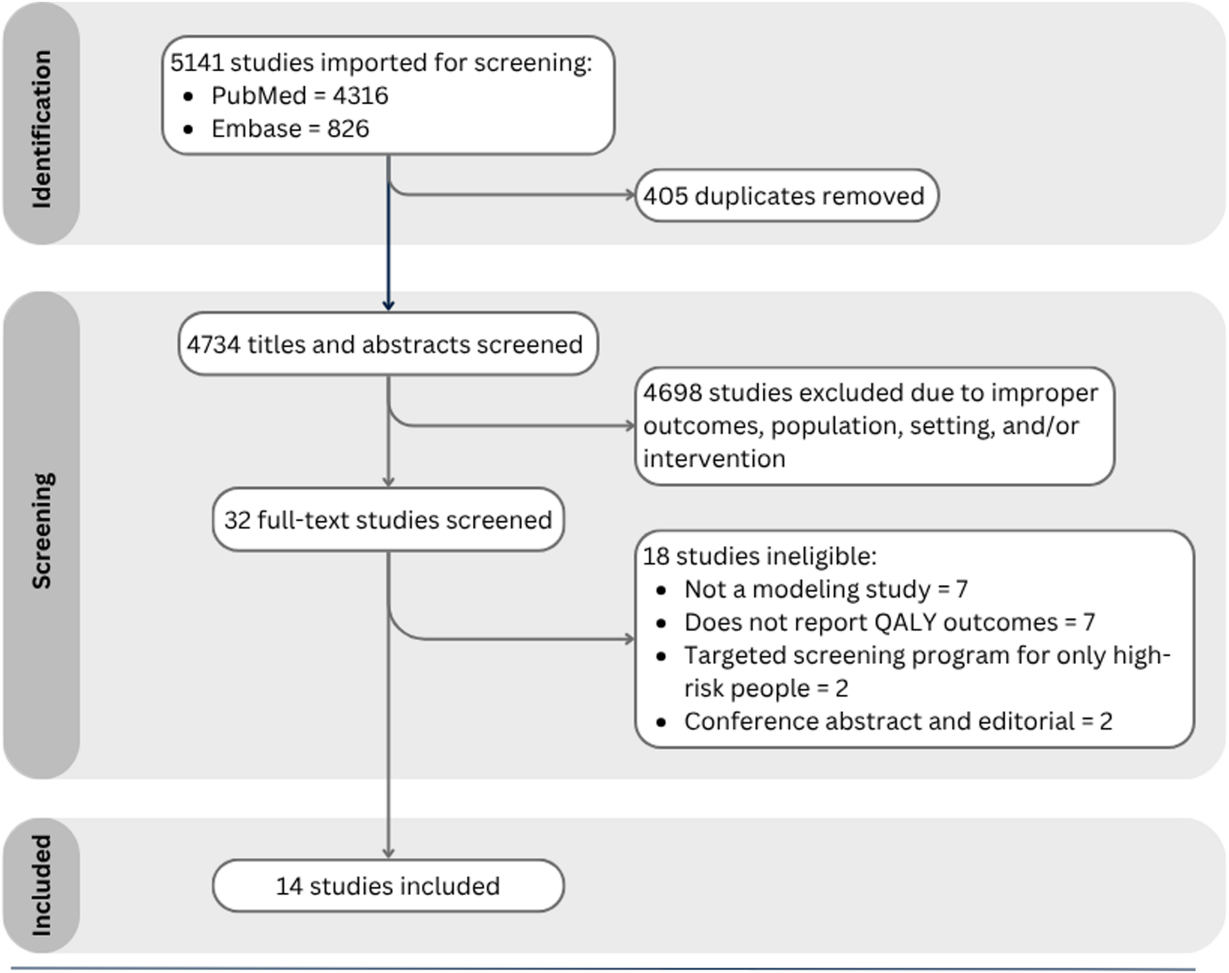
PRISMA flowchart of selected studies. Flowchart of decision making regarding the final studies to include in the systematic review. Titles and abstracts were excluded if they did not meet our inclusion criteria in terms of: outcome (e.g., studies on non-tuberculous mycobacteria, vaccine development, or immunological diagnostics such as interferon-gamma release assay or tuberculin skin test); population (e.g., studies focusing on healthcare workers, close contacts, or the general population instead of migrants); setting (e.g., studies conducted in high TB incidence countries); and intervention/design (e.g., non-modeling studies or studies not evaluating TB preventive treatment or its consequences). QALY = quality-adjusted life year

The search identified 5,141 records. After screening, 32 studies were selected for full-text review and 14 met the inclusion criteria (Figure 1). Of the 14 included studies, six were conducted in the United States,^13–15,17–19^ two in Canada,^11,20^ and one each in Sweden,^10^ Germany,^21^ Singapore,^22^ Turkey,^23^ Oman,^24^ and Australia.^12^ Most studies were published after 2015. The majority of studies (8/14) employed Markov modeling,^10,12–14,17,22–24^ and all studies used a time horizon of at least 10 years (Table 1). All studies used a health system perspective (Hsieh et al^19^ also did a societal perspective) and most a 3% discount rate (Supplementary Data Table S3). All included studies included TBI testing prior to TPT. Ten considered TST,^10–14,17,20,23–25^ while 12 considered IGRA.^10–14,17–22,24^ With respect to regimens, nine studies considered mono-isoniazid regimens (six or nine-months of isoniazid),^11,12,14,17,20,22–25^ eight studies considered three months of once-weekly isoniazid and rifapentine,^12,13,17–19,22–24^ seven studies considered four months of rifampin,^10–12,20–22,24^ and two studies considered 3 months of isoniazid and rifampin^10,22^ **(**Supplementary Data Table S4).

**Table 1.** Characteristics of included studies.

Author (year)	Country	Immigrant group modeled	Modeling method	Time horizon (years)	Age distribution modeled	TB infection prevalence
Al Abri (2020)	Oman	Newly arrived migrants	Markov model	Lifetime	20 years	43%
Campbell (2017)	Canada	Newly arrived permanent residents	Discrete event simulation	10	Based on demographics of a reference cohort of permanent residents in Canada in 2014	6.41–36.59% By country of origin
Campbell (2019)	Canada	Newly arrived permanent residents	Discrete event simulation	25	Based on demographics of a reference cohort of permanent residents in Canada in 2014	1.59–31.62% By country of origin
Dale (2021)	Australia	Newly arrived migrants	Markov model	Lifetime	Seven age groups, as young as 11 years old	17–32.4% By age group and country of origin
Goodell (2019)	USA	Current and new non-US born individuals	Markov model	64	Fourteen age groups, as young as 15 years old	19.4% varied by risk group
Hsieh (2025)	USA	Newly arrived migrants	Discrete event simulation	Lifetime	Mean age 29	12.4%
Ilaiwy (2021)	Turkey	Refugees	Markov model	20	Fourteen age groups, as young as 15 years old. Mean age 30	16.3%
Jo (2021)	USA	Current non-US born individuals	Dynamic Transmission Model	30	Based on the 5-year estimates from the American Community Surveys of 2005 and 2014	24–43%
Lim (2021)	Singapore	Migrants immigrating <1 year prior	Markov model	50	20–62 years and specific risk group	22.1% varied by age group
Linas (2011)	USA	Recent immigrants and those arriving <5 years prior	Markov model	Lifetime	Five age groups, as young as 6 years old	2.8–43.7% By age and risk groups
Marx (2021)	Germany	Asylum seekers	Probabilistic decision-analytic model	20	15–34 years	17.5%
Porco (2006)	USA	Newly arrived immigrants	Dynamic Transmission Model	20	Mean age 53.3	47%
Shedrawy (2021)	Sweden	Newly arrived migrants	Markov model	50	Seven age groups, as young as 0 years old up to ≥55 years old	25%
Tasillo (2017)	USA	Non-US born residents	Markov model	Lifetime	Specific to risk group, aged from 30 to 70 years	15.9%

### Quality assessment summary

No study was completely missing key quality criteria and quality scores ranged from 66% to 100% (Table 2). Most parameter sources were derived from systematic reviews, local data, or high-quality randomized trials. However, six studies did not consider important events such as risks and consequences of TPT-related adverse events.^10,17,19,22–24^ Only a minority used primary data to estimate health utility; nine relied on assumptions, most commonly around the impact of adverse events and hospitalization.^11,13,14,17,18,20–22,24^

**Table 2.** Quality assessment.

Study	Model considers all relevant events and processes?	Model rigorously sources all relevant parameters?	Health state utilities used are reflective of the population modeled and based on evidence?	Overall score
	(points = 8)	(points = 5)	(points = 3)	(Max = 16)
Al Abri (2020)	Somewhat	Yes	Somewhat	10.5 (66%)
Campbell (2017)	Yes	Yes	Somewhat	14.5 (91%)
Campbell (2019)	Yes	Yes	Somewhat	14.5 (91%)
Dale (2021)	Yes	Yes	Yes	16 (100%)
Goodell (2019)	Somewhat	Yes	Somewhat	10.5 (66%)
Hsieh (2025)	Somewhat	Yes	Yes	12 (75%)
Ilaiwy (2021)	Somewhat	Yes	Yes	12 (75%)
Jo (2021)	Yes	Yes	Somewhat	14.5 (91%)
Lim (2021)	Somewhat	Yes	Somewhat	10.5 (66%)
Linas (2021)	Yes	Yes	Somewhat	14.5 (91%)
Marx (2021)	Yes	Yes	Somewhat	14.5 (91%)
Porco (2006)	Yes	Yes	Yes	16 (100%)
Shedrawy (2021)	Somewhat	Yes	Yes	12 (75%)
Tasillo (2017)	Yes	Yes	Somewhat	14.5 (91%)

Note: ‘Yes’ received full points, ‘Somewhat’ received half points, and ‘No’ received no points.

### Risks and consequences associated with TPT

**Figure 2.**
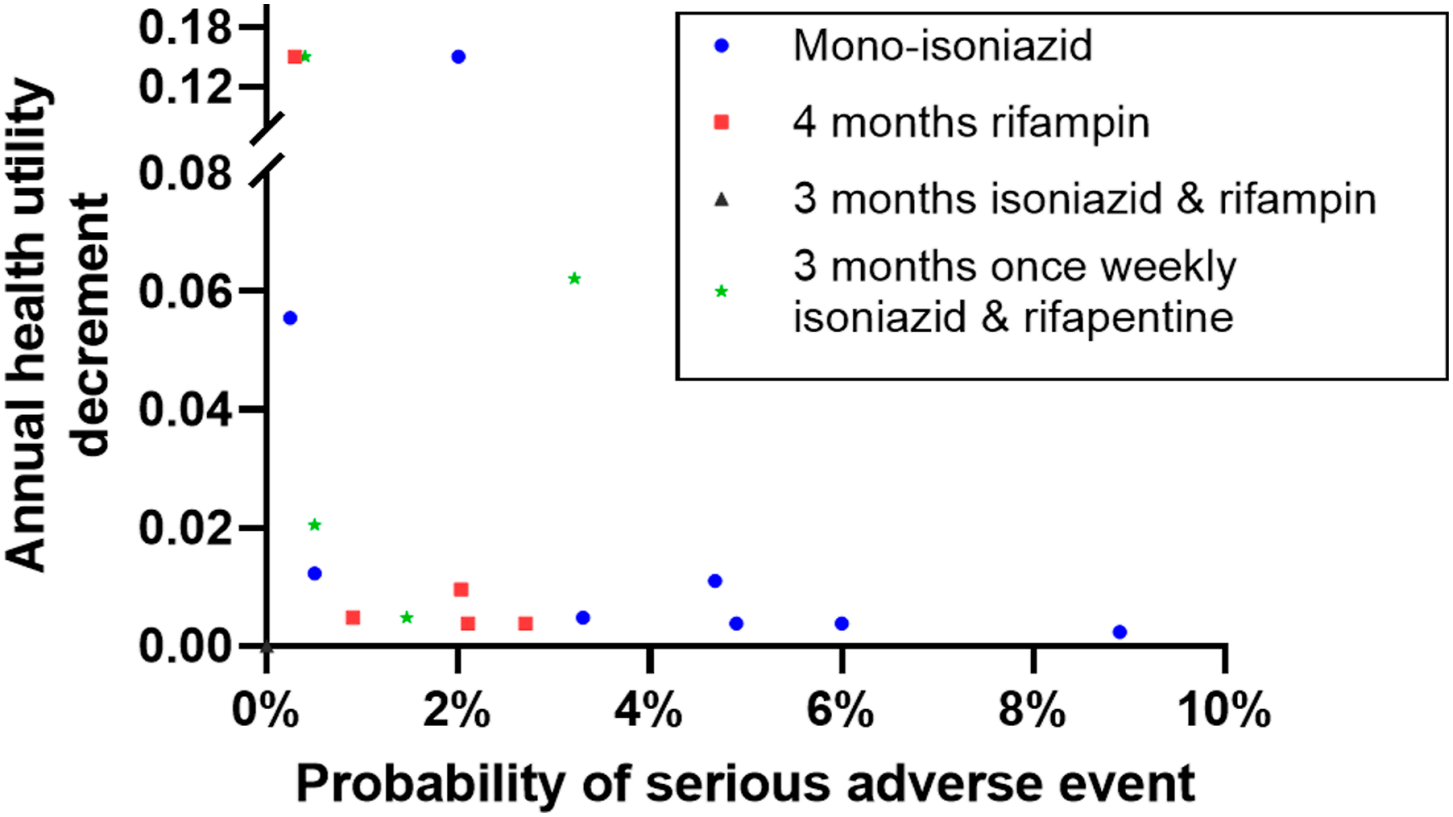
Distribution of risks for major adverse events and their attendant impacts on annual health disutility, stratified by regimen. Comparison of probability of major adverse events (x-axis) and annual health disutility associated with each event (y-axis) by regimen modeled in included studies. Note that 3-months isoniazid and rifampin is at the origin (0,0).

No study assumed health disutility from TBI alone. Two studies^19,24^ included minor health disutility attributed to the experience of receiving TPT (Supplementary Data Table S5). Major adverse events associated with TPT were modeled in 12 studies^10–14,17,18,20–22,24,25^ and most often defined as hepatotoxicity, but also including broader definitions such treatment discontinuation or hospitalization (Supplementary Data Table S6). Estimated probabilities for major adverse events ranged from 0% to 6%, and varied by regimen (Figure 2). Eight studies provided enough detail to estimate disutility from major adverse events^11–14,18,20,21,25^ (Table 3), with an average annual disutility (i.e., QALYs lost) of 0.017 (range: 0.0038–0.0625; coefficient of variation: 1.18). Most studies assumed a duration of 7 days to 1 month, though one extended the impact over the full treatment course (3 months). No study explicitly used the term ‘minor’ adverse events when discussing risks of TPT, though some considered ‘non-hepatitis’ adverse event risks. Six studies^11,12,18,20,21,25^ considered the probability of hospitalization from TPT-related adverse events, with probabilities ranging from 0.008% to 0.5% (mean: 0.049%) and an average annual disutility of 0.00759. Eight studies modeled the probability of death due to major adverse events related to TPT, with estimates ranging from 0% to 0.04%^11–14,20–22,25^ (Supplementary Data Table S5).

**Table 3.** Consequences associated with TB disease and adverse events (AEs) with TB preventive treatment (TPT).

Author (year)	Healthy utility^A^	TB disease			AEs with TPT		
		TB utility	Duration of TB impact	Annual disutility from TB	AE disutility	Duration of AE impact	Annual disutility from AE
Al Abri (2020)	1	0.8	1 year	0.2	0.15	Unclear	Unclear
Campbell (2017)	0.81	0.69	1 year	0.12	0.2	7 days	0.0038
Campbell (2019)	0.81	0.69	1 year	0.12	0.2	7 days	0.0038
Goodell (2019)	NS	NS	NS	0.046	NC	NC	NC
Hsieh (2025)	1	0.92	6 months	0.04	NC	NC	NC
Ilaiwy (2021)	0.9	0.76	1 year	0.14	NC	NC	NC
Lim (2021)	1	0.827	6 months	0.0865	0.75	NS	Unclear
Porco (2006)	1	0.45	4 months	0.15	0.265	1 month	0.022
Linas (2011)	1	0.8	6.4 months	0.11	0.15	1 month	0.0125
Dale (2021)	0.8733	0.7998	9 months	0.0551	0.25	7 days	0.0048
Marx (2021)	1	0.67	6 months	0.165	0.25	7 days	0.0048
Shedrawy (2021)	NS	NS	6 months	0.14	NC	NC	NC
Jo (2021)	NS	NS	NS	0.12	0.25	3 months	0.0625

A If the model only reported TBI utility, we used this value.

NS = not stated; NC = not considered.

### Risks and consequences associated with TB disease

All studies considered health disutility associated with TB disease. When standardized to an annual scale, disutility (i.e., QALYs lost) ranged from 0.04 to 0.2 (mean: 0.11, coefficient of variation: 0.4). The duration of impact on health disutility associated with TB disease was not standardized across studies, ranging from 4-12 months. Most commonly, studies considered the impact of TB to last the duration of treatment (typically 6 months), however some studies assumed no disutility soon after treatment initiation, while others attributed disutility for an entire year (Table 3). None of the included studies considered major adverse events as an outcome directly attributable to TB disease treatment; only one study embedded minimal health disutility associated with major adverse events in its estimate of TB utility. This study considered 0.7% of individuals would have a major adverse event and experience a loss of 0.25 QALYs for 1 week.^12^ Hospitalization during TB disease was often acknowledged through cost, but rarely linked explicitly to health utility loss (Supplementary Data Table S7). All but one study^25^ incorporated the risk of TB-related death, with probabilities ranging from 0% to 7%, with an average of 5.3% (Supplementary Data Table S7). Three studies considered post-TB consequences in their primary analysis.^17,19,23^ One study considered both morbidity and mortality, estimating a loss of 0.125 QALYs post-TB.^17^ The other two estimated annual post-TB morbidity-related disutility of 0.01^19^ and 0.053.^23^ Two additional studies included post-TB consequences only in sensitivity analyses.^12,14^

### Sources of health utility estimates

Sources for health disutility were inconsistently reported. One study leveraged previous research they conducted in their population of interest to parameterize health utilities,^10^ while many cited prior cost-effectiveness analyses, rather than primary data (Supplementary Data Table S8). For TB disease disutility, 10 different primary sources^26–35^ were cited across the 14 studies. However, even among studies referencing the same source, disutility estimates varied (Table 3, Supplementary Data Table S8). In general, this appeared to be due to differences in assumptions about the duration of impact of TB disease and assumptions surrounding ‘baseline’ health utility (i.e., health utility in absence of TB). Disutility associated with adverse events and hospitalization were commonly assumed. Although some studies which primarily considered the adverse event of hepatotoxicity with TPT used estimates of disutility associated with hepatitis (n=3 studies).^12,13,22^

## DISCUSSION

In this systematic review, we found substantial variability in how cost-utility analyses of TBI screening and treatment among immigrants considered the risks and consequences of TB disease and TPT. Notably, there was wide variation in how studies incorporated the risks of major adverse events related to TPT and their corresponding impact on health utility. Though many studies used similar sources to parameterize health utilities in their model, variability remained.

As TB disease is the outcome that TPT aims to prevent, the way its health utility impact is modeled is especially important. However, we found significant variation in both the utility values used to represent TB disease and the duration of impact. While it may be reasonable to expect different elicited TB utilities across tools and settings, all studies were done in high-income settings in similar populations and even studies from the same country had varying estimates of the impact of TB on health utility.^13,14,17–19,25^ We observed inconsistencies in both the length of time individuals were modeled to remain in the TB health state and various parameterizations of the utility parameter for ‘healthy’ health states. These variations may strongly influence the estimated benefits of averted TB disease and the cost-effectiveness of TPT. Further, it was not always clear how utility estimates were derived in each study, particularly when multiple sources were cited. How utility estimates are arrived at, who the comparator ‘healthy’ population is, and how utility estimates are modelled must be adequately reported and justified in future models. We expect a more thoughtful reporting of these important parameters to improve the consistency of estimates across settings.

Few longitudinal studies, notably by Marra et al., Bauer et al., and Shedrawy et al., have evaluated health utility trajectories among people treated for TB disease, but available data suggest utility is not static during treatment and improves within the first few months of treatment.^31,33,35^ Despite this, most included studies modeled TB-related disutility as a constant throughout treatment. Future economic models should treat the TB health state as dynamic with transparent reporting of how utility parameters were estimated.^12^ Another important but underrepresented consequence of TB disease is adverse events associated with treatment. Randomized trials suggest that approximately 10% of adults experience a serious adverse event during treatment,^36^ which are likely to impact health utility. However, only Dale et al. explicitly modeled the health utility impact of these events, and used a probability much lower than 10%.^12^ Given that most utility estimates used in the included models were derived from cross-sectional studies, it is unlikely that these adverse events were captured in baseline utility values.

There is growing recognition of the prevalence of post-TB morbidity among TB survivors and its impact on quality of life.^37^ In burden of disease studies, the number of disability-adjusted life years caused by post-TB morbidity is equivalent to those caused by TB disease itself.^38^ Despite its importance, post-TB morbidity was rarely modeled, and when included, the estimates of health disutility varied widely. While further research is needed to understand quality of life among TB survivors, existing disability weights can provide a reasonable interim proxy.^38^

The most consequential risk associated with TPT are adverse events. Despite this, two of the reviewed studies (Ilaiwy et al. and Hsieh et al.) did not account for them, potentially leading to an overestimation of the benefits of TPT.^19,23^ Among those that did, definitions varied widely, resulting in divergent estimates of adverse event risk, even for the same regimen. Hepatitis was the most frequently modeled adverse event, despite being relatively rare with rifamycin-based TPT (now considered the new gold standard for TPT), where other events like systemic drug reactions are more common and of concern.^39^ Future models should adopt more comprehensive and standardized definitions of adverse events, such as events leading to treatment discontinuation.^40,41^ Minor adverse events, such as rash, nausea, dizziness, headache, and body pain, are frequently reported in TPT trials, with up to 75% of participants experiencing at least one symptom.^39,42^ However, none of the reviewed models considered these events explicitly. Although individually minor, their high prevalence may lead to cumulative impacts on population-level utility estimates. Other factors such as pill burden or anxiety related to diagnosis,^43^ may also impact health utility, but were only broadly considered by Al Abri et al. in primary analyses.^24^

Our findings reflect broader challenges previously noted in the literature on health economic modeling for TB.^44–46^ A major recurring issue is the lack of consistency and transparency in how utility values and adverse event probability were derived or applied, limiting the reproducibility of findings and the ability to meaningfully compare results across studies. Consistent terminology, standardized definitions for adverse events, and detailed reporting of parameter sources and justifications are needed.

The major strength of this review is its detailed and systematic appraisal of utility-related inputs in TB economic models. We examined models in detail and systematically evaluated specific model parameters and primary sources for key health utility parameters. Our analysis has highlighted key areas where more consistency is needed, such as adverse events, and where future research may be influential, such as on the impact of post-TB sequelae. However, our review has limitations. We only compared risks and consequences associated with TPT and TB disease between models, and did not endeavor to also compare other parameters such as those associated with cascades of care and costs, which are more variable across settings.^47^ We also did not attempt to correlate the different parameterization of risks and consequences between models with cost-effectiveness outcomes. Given the numerous parameters that can affect these outcomes, such as age and TBI prevalence, it was outside the scope of this review. Finally, our review focused specifically on models assessing TBI screening and treatment among immigrants to low-incidence countries. We selected these models as it is an area of controversy with differing conclusions around cost-effectiveness. An assessment of their parameterization may promote more consistency in modeling decisions.

## CONCLUSIONS

Our systematic review revealed considerable variability in how key parameters for the risks and consequences of TB disease and TPT were defined in cost utility analyses. These methodological inconsistencies are likely to contribute to divergent cost-effectiveness estimates of TBI screening and treatment programs for immigrant populations. To improve the quality and comparability, greater standardization and transparency for health utility parameters are needed to improve interpretability and policy relevance.

## Supplementary Material


