# Supplementary material for: Risks and consequences of TB and its prevention in cost-utility analyses among immigrants: a systematic review
Source: IJTLD Open. 2025 Oct 10;2(10):555–62. doi: 10.5588/ijtldopen.25.0355 (PMC12517258; doi:10.5588/ijtldopen.25.0355)
Supplement: Supplementary file 1 [file ijtldopen25-0355_supplementarydata1.pdf]

SUPPLEMENTAL MATERIAL

Risks and consequences of tuberculosis and its prevention in cost-utility analyses among immigrants: A systematic review

Table of Contents

Supplemental tables 2
Table S1.1. EMBASE Search 2
Table S1.2. PUBMED Search 2
Table S2. Fields Extracted from Included Studies 3
Table S3. Study perspective and discount rate 4
Table S4. Intervention Evaluated 4
Table S5. Risks associated with Tuberculosis Infection and Its Treatment 5
Table S5.1 Consequences associated with Tuberculosis Infection and Its Treatment 6
Table S6. Adverse events definitions for tuberculosis preventive treatment 7
Table S7. Risks associated with Tuberculosis Disease and Its Treatment 8
Table S7.1 Consequences associated with Tuberculosis Disease and Its Treatment 8
Table S8. Sources for health utility estimates in each model 9

Supplemental tables

Table S1.1. EMBASE Search

Search Number	Term
1	exp latent tuberculosis/
2	(latent adj3 tuberculosis).mp.
3	(latent adj3 tb).mp.
4	ltbi.mp.
5	(tuberculosis adj2 infection).mp.
6	(tb adj2 infection).mp.
7	tuberculosis prevention.mp.
8	tb prevention.mp.
9	1 or 2 or 3 or 4 or 5 or 6 or 7 or 8
10	exp economic evaluation/
11	cost-utility.mp.
12	cost analysis.mp.
13	cea.mp.
14	exp quality adjusted life year/
15	qaly.mp.
16	exp disability adjusted life year/
17	daly.mp.
18	cost-effectiveness.mp.
19	or/10-18
20	9 and 19
21	limit 20 to yr="2000-current"

Table S1.2. PUBMED Search

Field	Variables Extracted
1	((latent tuberculosis)
2	OR (latent tb)
3	OR (tuberculosis infection)
4	OR (tb infection)
5	OR (TB prevention))
6	AND ((cost-utility)
7	OR (cost-effectiveness)
8	OR (quality adjusted life year)
9	OR (QALY)
10	OR (cost analysis)
11	OR (CEA))

Table S2. Fields Extracted from Included Studies

Field	Variables Extracted
**STUDY CHARACTERISTICS**	
Study-Identifiers	Study ID, First Author, Year Published, Title, Abstract, Aim, Main Population Studied.
Study Design	Modeling method, Time horizon, Study location, The Discount Rate, TB Infection Prevalence, Probability of Developing TB Disease, QALY Value for a Healthy Individual,
Test Used	Tuberculin Skin Test, IGRA Name, TB Antigen Skin Test; Sensitivity, Specificity (BCG Vaccination Considered for Tuberculin Skin Test), Completion Rate; Timing of Testing After Arrival, Population Screening Coverage, Proportion Recommended Treatment, Proportion Accepting Treatment.
Regimen Used	9 Months Daily Isoniazid, 6 Months Daily Isoniazid, 4 Months Daily Rifampin, 3 Months Daily Isoniazid and Rifampin, 3 Months Weekly Isoniazid and Rifapentine, 1 Month Daily Isoniazid and Rifapentine; Completion Rate, Efficacy Among Completers.
**POPULATION CHARACTERISTICS**	
Patients Included	Migrants to Low-Incidence Countries Including Refugees and Asylum Seekers, Close Contacts of Tuberculosis Cases.
Age	Age Distribution (Mean Age or Age Groups Modeled).
HIV Information	Proportion Infected, Impact, or Subgroup if Applicable.
Chronic Kidney Disease Information	Proportion on Dialysis or With Chronic Kidney Disease, Impact, or Subgroup if Applicable.
Diabetes Information	Proportion With Diabetes, Impact, or Subgroup if Applicable.
Cancer Information	Proportion With Cancer, Impact, or Subgroup if Applicable.
Organ or Hematopoietic Transplantation Information	Proportion With Transplantation, Impact, or Subgroup if Applicable.
Immune Suppressing Medication Information	Proportion Receiving Immune Suppressing Medication, Impact, or Subgroup if Applicable.
BCG vaccination	BCG Vaccination Considered, Proportion Vaccinated With BCG, Impact of BCG Vaccination on Test Performance.
**OUTCOMES**	
Risks of Tuberculosis Disease and Its Treatment	Tuberculosis Disease Considered; Serious Adverse Events, Hospitalization, Post-Tuberculosis Consequences, Death; Risk Estimation, Health Utility, Duration of Each Risk Impact, Annual Disutility.
Risks of TB Infection and Its Treatment	Tuberculosis Infection and Preventive Treatment Considered; Major and Minor Adverse Events, Hospitalization, Death; Risk Estimation, Health Utility, Duration of Each Risk Impact, Annual Disutility.
Study outcomes	QALYs Added per Person by Each Intervention Strategy.

Table S3. Study perspective and discount rate

Author (year)	Perspective	Discount rate
Al Abri (2020)	Health system	0%
Campbell (2017)	Health system	1.5%
Campbell (2019)	Health system	3%
Dale (2021)	Health system	3%
Goodell (2019)	Health system	3%
Hsieh (2024)	Health system and societal	3%
Ilaiwy (2021)	Health system	3%
Jo (2021)	Health system	3%
Lim (2021)	Health system	3%
Linas (2011)	Health system	3%
Marx (2021)	Health system	3%
Porco (2006)	Health system	3%
Shedrawy (2021)	Health system	3%
Tasillo (2017)	Health system	3%

Table S4. Intervention Evaluated

Author (year)	TB infection tests used		Regimen used				
	TST	IGRA	9H	6H	4R	3HR	3HP
Al Abri (2020)	x	x		x	x		x
Campbell (2017)	x	x	x		x		
Campbell (2019)	x	x	x		x		
Dale (2021)	x	x	x	x	x		x
Goodell (2019)	x	x	x	x			x
Hsiehet (2024)		x					x
Ilaiwy (2021)	x		x				x
Jo (2021)		x					x
Lim (2021)		x	x	x	x		x
Linas (2011)	x	x	x				
Marx (2021)		x			x		
Porco (2006)	x		x	x			
Shedrawy (2021)	x	x			x	x	
Tasillo (2017)	x	x					x

Abbreviations: TB, tuberculosis; TST, tuberculin skin test; IGRA, interferon-gamma release assay; 9H, 9 months of daily isoniazid; 6H, 6 months of daily isoniazid; 4R, 4 months of daily rifampin; 3HR, 3 months of daily isoniazid and rifampin; 3HP, 3 months of once weekly isoniazid and rifapentine.

Table S5. Risks associated with Tuberculosis Infection and Its Treatment

Author (year)	TPT risks			
**Regimen**	**Major AE(s)**	**Minor AE(s)**	**Hospitalization**	**Death**
**INH**				
Al Abri (2020)	0.02	NC	NC	NC
Campbell (2017)	0.06	NC	0.0006*	0.00000988
Campbell (2019)	0.049[1]	NC	0.00049*	0.00000988
Dale (2021)	0.010-0.055[9]	NC	0.000998-0.005059[12]	0 - 0.0006745[21]
Goodell (2019)	0.002; 0.0075[2]	NC	NC	NC
Ilaiwy (2021)	NC	NC	NC	NC
Lim (2021)	0.012; 0.018[3]	NC	NC	0.000012; 0.000018*
Porco (2006)	0.0458-0.0478[8]	NC	0.00008-0.00028[4]*	0.00001-0.000035[7]*
Linas (2011)	0.001–0.01[5]	NS	NS	0.00001-0.0001[13]*
**4R**				
Al Abri (2020)	0.003	NC	NC	NC
Campbell (2017)	0.027	NC	0.00027*	NS
Campbell (2019)	0.021	NC	0.00021*	NS
Dale (2021)	0.009[6]	NC	0.00030487[6]	0.0000060997[6]
Lim (2021)	0.003	NC	NC	0.000003*
Marx (2021)	0.0203	NC	0.0003	NC
**3HR**				
Lim (2021)	0.005	NC	NC	0.000005*
Shedrawy (2021)	0	NS	NC	NC
**3HP**				
Al Abri (2020)	0.004	NC	NC	NC
Dale (2021)	0.0033 - 0.026[8]	NC	0.001 - 0.003[10]	0 - 0.0004[11]
Hsieh (2024)	NC	NC	NC	NC
Ilaiwy (2021)	NC	NC	NC	NC
Jo (2021)	0.03215	NC	0.00015	NC
Tasillo (2017)	0.005	NC	NC	0.000005*

Abbreviations: TPT, tuberculosis preventive treatment; INH, isoniazid; 4R, 4 months of daily rifampin; 3HR, 3 months of daily isoniazid and rifampin; 3HP, 3 months of once weekly isoniazid and rifapentine; AE, adverse event; NC: Not considered (not mentioned at all); NS: Not specified (mentioned, but no value given); *Contingent values: for an explanation of how each of these was calculated;[1]0.00008 for 15–34 years, 0.00021 for 35–64 years, 0.00028 for 65 years and older; [2]0.002 for severe hepatitis, 0.0075 for mild hepatitis; [3]0.012 for 6H, 0.018 for 9H; [4]Mayor AE sufficient to warrant discontinuation, stratified by hepatitis and non-hepatitis events: 0.0458 for 15–34 years, 0.0471 for 35–64 years, 0.0478 for 65 years and older; [5]0.001 for 0-34 years, 0.01 for 35 years and older; [6]All ages; [7]0.00001 for 15–34 years, 0.000026 for 35–64 years, 0.000035 for 65 years and older; [8]0.0033 for ≤35-50 years, 0.026 for 51>65 years; [9]0.010 for 10-34 years, 0.026 for 35-64 years, 0.055 for ≥ 65 years; [10]0.001 for ≤35-50 years, 0.003 for 51>65 years; [11]0 for ≤35 years, 0.00013332 for 36-50 years, 0.0004 for 51>65 years; [12]0.001307 for 0-17 years, 0.000998 for 18-34 years, 0.001847 for 35-64 years, 0.005059 for >65 years; [13]0 for 0-34 years,0.0002462 for 35-64 years, 0.0006745 for >65 years; [21]0.00001 for 0-34 years, 0.0001 for 35 years and older

Table S5.1 Consequences associated with Tuberculosis Infection and Its Treatment

Author (year)	Annual Disutility		
	Major AE(s)	Hospitalization	TPT itself
Al Abri (2020)	Unclear	NC	0.01
Campbell (2017)	0.0038	0.0059	0
Campbell (2019)	0.0038	0.0059	0
Dale (2021)	0.0048	NS	0.0133*
Goodell (2019)	NC	NC	0
Hsieh (2024)	NC	NC	0.0005
Ilaiwy (2021)	NC	NC	0.045*
Jo (2021)	0.0625	NS	0
Lim (2021)	Unclear	NC	0
Linas (2011)	0.0125	NS	0.1*
Marx (2021)	0.0048	0.009589	0
Porco (2006)	0.022	0.009	0
Shedrawy (2021)	NC	NC	0
Tasillo (2017)	0.02054	NS	0.1*

Abbreviations: AE, adverse events; NC: Not considered (not mentioned at all); NS: Not specified (mentioned, but no value given); *Value from sensitivity analysis.

Table S6. Adverse events definitions for tuberculosis preventive treatment

Author (year)	Adverse events definitions
Al Abri (2020)	Hepatotoxicity
Campbell (2017)	Any adverse event that leads to the discontinuation of TPT or death due to fatal hepatotoxicity
Campbell (2019)	Any adverse event that leads to the discontinuation of TPT or death
Dale (2021)	Severe adverse event defined as grade 3–5
Goodell (2019)	Mild and severe hepatitis
Hsieh (2024)	NC
Ilaiwy (2021)	NC
Jo (2021)	Mild and severe treatment toxicity that may lead to hospitalization
Lim (2021)	Hepatitis and fatal hepatitis
Linas (2011)	Nonfatal or fatal hepatitis and other adverse events that may lead to the discontinuation of TPT for reasons other than toxicity (e.g., nonadherence)
Marx (2021)	TPT drug-toxicity events that may lead to hospitalization
Porco (2006)	Non-hepatitis or Hepatitis events that may lead to hospitalization or death
Shedrawy (2021)	NC, but mentioned patients reported nausea, dizziness, and body pain
Tasillo (2017)	Hepatotoxicity that may lead to hospitalization

TPT, tuberculosis preventive treatment; AE, adverse event; NC: Not considered (not mentioned at all)

Table S7. Risks associated with Tuberculosis Disease and Its Treatment

Author (year)	TB risks			
	Major AE(s)	Hospitalization	Death	Post TB consequences
Al Abri (2020)	NC	NC	0.07	NC
Campbell (2017)	NC	NS	0.0476	NC
Campbell (2019)	NC	NS	0.0476	NC
Dale (2021)	0.0007	NC	0.05[1]	0
Goodell (2019)	NC	NC	0.047[5]	1.00
Hsieh (2024)	NC	NC	0.0544	1.00
Ilaiwy (2021)	NC	NC	0.069	1.00
Jo (2021)	NC	0.49	0.058[4]	NC
Lim (2021)	NC	NC	0-0.1[1]	NC
Linas (2011)	NC	0.45-0.63[6]	0.05–0.06[3]	0
Marx (2021)	NC	NC	0.015	NC
Porco (2006)	NC	0.081–0.66[2]	NS	NC
Shedrawy (2021)	NC	NC	0.07	NC
Tasillo (2017)	NC	0.503	0.05	0

Abbreviations: TB, tuberculosis; AE, adverse event; NC: Not considered (not mentioned at all); NS: Not specified (mentioned, but no value given); [1]Overall estimate, but modeled as age specific; [2]0,35 active smear positive, 0.081 active smear negative, 0.66 passive smear positive, 0.51 passive smear negative; [3]0.05 with no medical comorbidities, 0.06 with chronic conditions; [4]0.092 to HIV; [5]From Miller et al (Miller TL, McNabb S, Hilsenrath P, Pasipanodya J, Weis SE. Personal and societal health quality lost to tuberculosis. PLoS One. 2009;4:e5080. pmid:19352424; [6]0.45 for people without HIV and 0.63 for people with HIV.

Table S7.1 Consequences associated with Tuberculosis Disease and Its Treatment

Author (year)	Annual Disutility			
	TB	Major AE(s)	Hospitalization	Post TB consequences
Al Abri (2020)	0.2	NC	NC	NC
Campbell (2017)	0.12	NC	NS	NC
Campbell (2019)	0.12	NC	NS	NS
Dale (2021)	0.0551	0.0048†	NC	0.024*
Goodell (2019)	0.046	NC	NC	0.125
Hsieh (2024)	0.04	NC	NC	0.01
Ilaiwy (2021)	0.14	NC	NC	0.053
Jo (2021)	0.12	NC	NS	NS
Lim (2021)	0.0865	NC	NC	NC
Linas (2011)	0.11	NC	NS	0.1*
Marx (2021)	0.165	NC	NC	NC
Porco (2006)	0.15	NC	NS	NC
Shedrawy (2021)	0.14	NC	NC	NC
Tasillo (2017)	0.11	NC	NS‡	0.13*

Abbreviations: AE, adverse events; TB, tuberculosis; NC: Not considered (not mentioned at all); NS: Not specified (mentioned, but no value given); *Value from sensitivity analysis; †embedded within the tuberculosis disutility, not considered separately. ‡no explicit disutility associated with hospitalization but those hospitalized experienced longer treatment and therefore greater disutility.

Table S8. Sources for health utility estimates in each model

Study	Tuberculosis health utility	Adverse event health utility	Hospitalization health utility	Post-tuberculosis health utility	Tuberculosis infection health utility	Tuberculosis preventive treatment health utility
Al Abri (2020)	**Unclear how value derived from included studies**<br>- Dion et al, Qual Life Res. 2004;13:653-65.<br>- Bauer et al, Qual Life Res. 2015;24:1337-49.	**Unclear how value derived from included studies**<br>- Dion et al, Qual Life Res. 2004;13:653-65.<br>- Bauer et al, Qual Life Res. 2015;24:1337-49.	**Unclear how value derived from included studies**<br>- Dion et al, Qual Life Res. 2004;13:653-65.<br>- Bauer et al, Qual Life Res. 2015;24:1337-49.	N/A	**Unclear how value derived from included studies**<br>- Dion et al, Qual Life Res. 2004;13:653-65.<br>- Bauer et al, Qual Life Res. 2015;24:1337-49.	**Unclear how value derived from included studies**<br>- Dion et al, Qual Life Res. 2004;13:653-65.<br>- Bauer et al, Qual Life Res. 2015;24:1337-49.
Campbell (2017)	-Bauer et al, Qual Life Res. 2015;24:1337-49.**<br>-Guo et al, Value Health. 2008;11:1154-61.<br>-Marra et al, Chest. 2008;133:396-403.	**Assumption**<br>(Holland et al, Am J Respir Crit Care Med. 2009;179(11):1055-1060.)	**Assumption**<br>(Holland et al, Am J Respir Crit Care Med. 2009;179(11):1055-1060.)	N/A	-Bauer et al, Qual Life Res. 2015;24:1337-49.**<br>-Guo et al, Value Health. 2008;11:1154-61.<br>-Marra et al, Chest. 2008;133:396-403.	N/A
Campbell (2019)	Bauer et al, Qual Life Res. 2015;24:1337-49.	**Assumption**<br>(Holland et al, Am J Respir Crit Care Med. 2009;179(11):1055-1060.)	**Assumption**<br>(Holland et al, Am J Respir Crit Care Med. 2009;179(11):1055-1060.)	N/A	**Assumption**	N/A
Dale (2021)	Bauer et al, Qual Life Res. 2015;24:1337-49.	McLernon et al. Med Decis Making. 2008;28(4):582-92.	N/A	Bauer et al, Qual Life Res. 2015;24:1337-49.	Bauer et al, Qual Life Res. 2015;24:1337-49.	Bauer et al, Qual Life Res. 2015;24:1337-49.
Goodell (2019)	Miller et al. PLoS One. 2009;4:e5080.	N/A	N/A	Miller et al. PLoS One. 2009;4:e5080.	N/A	N/A
Hsieh (2025)	Guo et al. Health Qual Life Outcomes 2009;7:14.	N/A	N/A	**Unclear how value was selected**<br>Menzies et al. Lancet Glob Health. 2021;9:e1679-87.	**Assumption**	**References Jo et al (2021), which ultimately uses the below reference, although unclear how the value was derived.**<br>Guo et al, Value Health. 2008;11:1154-61.
Ilaiwy (2021)	Guo et al, Value Health. 2008;11:1154-61.	N/A	N/A	-Guo et al, Value Health. 2008;11:1154-61.<br>-Quaife et al. Lancet Resp Med. 2020;8(4):332-333.**	Guo et al, Value Health. 2008;11:1154-61.	N/A
Jo (2021)	Guo et al, Value Health. 2008;11:1154-61.	**Assumption**<br>(Holland et al, Am J Respir Crit Care Med. 2009;179(11):1055-1060.)	**Assumption**<br>(Holland et al, Am J Respir Crit Care Med. 2009;179(11):1055-1060.)	N/A	N/A	**References another model (Holland et al), which ultimately uses the below reference, although unclear how the value was derived.**<br>Guo et al, Value Health. 2008;11:1154-61.

Study	Tuberculosis health utility	Adverse event health utility	Hospitalization health utility	Post-tuberculosis health utility	Tuberculosis infection health utility	Tuberculosis preventive treatment health utility
Lim (2021)	**References another model (Dobler et al) which references the below.**<br><br>Awaisu et al. Tob Induc Dis. 2012;10(1):2.	McLernon et al. Med Decis Making. 2008;28(4):582-92.	N/A	N/A	N/A	**Assumption**
Linas (2011)	**The same three references are used for all utility parameters and it is unclear how each are derived**<br><br>-Dion et al. Med Decis Making. 2002;22:S102-114.<br>-Dion et al. Qual Life Res 2004; 13:653-665.<br>-Marra et al. Chest. 2008;133:396-403.	**The same three references are used for all utility parameters and it is unclear how each are derived**<br><br>-Dion et al. Med Decis Making. 2002;22:S102-114.<br>-Dion et al. Qual Life Res 2004; 13:653-665.<br>-Marra et al. Chest. 2008;133:396-403.	N/A	**The same three references are used for all utility parameters and it is unclear how each are derived**<br><br>-Dion et al. Med Decis Making. 2002;22:S102-114.<br>-Dion et al. Qual Life Res 2004; 13:653-665.<br>-Marra et al. Chest. 2008;133:396-403.	N/A	**The same three references are used for all utility parameters and it is unclear how each are derived**<br><br>-Dion et al. Med Decis Making. 2002;22:S102-114.<br>-Dion et al. Qual Life Res 2004; 13:653-665.<br>-Marra et al. Chest. 2008;133:396-403.
Marx (2021)	**Uses disability weight to estimate lost health utility due to tuberculosis.**<br><br>-Vos et al. Lancet, 2020;396(10258):1204-1222.**<br>-Guo et al, Value Health. 2008;11:1154-61.	**Assumption**<br>(Holland et al, Am J Respir Crit Care Med. 2009;179(11):1055-1060.)	**Assumption**<br>(Holland et al, Am J Respir Crit Care Med. 2009;179(11):1055-1060.)	N/A	**Assumption**	N/A
Porco (2006)	**Unclear how value derived from included studies**<br><br>-Dion et al. Med Decis Making. 2002;22:S102-114.<br>-Dion et al. Qual Life Res 2004; 13:653-665.<br>-Schechter et al. Am J Prev Med. 1990; 6:167-175.	**References another study (Marchand et al), which then references the below study.**<br><br>Sackett et al. J Chronic Dis. 1978;31:697-704.	Sackett et al. J Chronic Dis. 1978;31:697-704.	N/A	N/A	N/A
Shedrawy (2021)	Shedrawy et al. IJTLD. 2020;24(4):461-63.	N/A	N/A	N/A	Shedrawy et al. Health Qual Life Outcomes. 2019;17(1):158	Shedrawy et al. Health Qual Life Outcomes. 2019;17(1):158
Tasillo (2017)	Guo et al, Value Health. 2008;11:1154-61.	McLernon et al. Med Decis Making. 2008;28(4):582-92.	N/A	N/A	**Assumption**	**Assumption**

**This is the study where the value appears to be derived from.
